# Feature selection leads to divergent neurobiological interpretations of brain-based machine learning biomarkers

**DOI:** 10.1038/s41562-026-02447-y

**Published:** 2026-04-15

**Authors:** Brendan D. Adkinson, Matthew Rosenblatt, Huili Sun, Javid Dadashkarimi, Link Tejavibulya, Corey Horien, Margaret L. Westwater, Raimundo X. Rodriguez, Stephanie Noble, Dustin Scheinost

**Affiliations:** 1 Yale School of Medicine, New Haven, CT USA; 2 Department of Biomedical Engineering, Yale University, New Haven, CT USA; 3 Department of Radiology, Perelman School of Medicine, Philadelphia, PA USA; 4 Department of Psychiatry, University of Pennsylvania, Philadelphia, PA USA; 5 Department of Radiology & Biomedical Imaging, Yale School of Medicine, New Haven, CT USA; 6 Department of Psychiatry, University of Oxford, Warneford Hospital, Oxford, UK; 7 Department of Bioengineering, Northeastern University, Boston, MA USA; 8 Department of Psychology, Northeastern University, Boston, MA USA; 9 Institute for Cognitive & Behavioral Health, Northeastern University, Boston, MA USA; 10 Department of Statistics & Data Science, Yale University, New Haven, CT USA; 11 Child Study Center, Yale School of Medicine, New Haven, CT USA; 12 Wu Tsai Institute, Yale University, New Haven, CT USA

**Keywords:** Cognitive neuroscience, Machine learning, Psychology, Computational neuroscience, Psychology

## Abstract

A central objective in human neuroimaging is to understand the neurobiology underlying cognition and mental health. Machine learning models trained on neuroimaging data are increasingly used as tools for predicting behavioural phenotypes, enhancing precision medicine and improving generalizability compared with traditional MRI studies. However, the high dimensionality of brain connectivity data makes model interpretation challenging. Prevailing practices rely on selecting features and, implicitly, interpreting identified feature networks as uniquely representative of a given phenotype while overlooking others. Despite its widespread use, how univariate feature selection balances the trade-off between simplification for optimizing modelling and oversimplification that misrepresents true neurobiology remains understudied. Here, using four large-scale neuroimaging datasets spanning over 12,000 participants and 13 outcomes, we demonstrate that edges discarded by feature selection can achieve significant prediction accuracies while yielding different neurobiological interpretations. These results are observed across cognitive, developmental and psychiatric phenotypes, extend to both functional connectivity (functional MRI) and structural (diffusion tensor imaging) connectomes, and remain evident in external validation. They suggest that focusing on only the top features may simplify the neurobiological bases of brain–behaviour associations. Such interpretations present only the tip of the iceberg when certain disregarded features may be just as meaningful, potentially contributing to ongoing issues surrounding reproducibility within the field. More broadly, our results reinforce that subtle brain-wide signals should not be ignored.

## Abstract (web summary)

Across more than 12,000 MRI scans, the authors show that features discarded during feature selection can rival top-ranked ones in predicting behaviour yet imply different brain circuits, warning that focusing only on top features can mislead neurobiology.

## Main

A central goal of human neuroimaging is to uncover the neurobiological underpinnings of cognition and mental health. Aligned with this objective, emerging machine learning approaches predict individual differences in behavioural phenotypes from brain structure and function^1–4^. These brain–behaviour predictive models reduce overfitting and provide more robust assessments of associations relative to traditional MRI studies by separating datasets into training and test samples^5–8^. They also offer opportunities to advance precision medicine strategies, ranging from illness subtyping to predicting individual treatment responses^9–11^. Accordingly, substantial efforts have been dedicated to improving model accuracy^12–14^, trustworthiness^15^, fairness^16,17^, reproducibility^18–23^ and generalizability^24–27^.

Neurobiological interpretability—the degree to which results can be mapped to known neural circuits and processes—is another key metric of model utility^28–30^. An easily interpretable model can uncover the neural circuits responsible for behaviour, even with modest prediction performance^31^. However, several challenges exist for interpreting machine learning models based on neuroimaging data^32–34^. To obtain more manageable representations of high-dimensional data, the field commonly employs univariate feature selection^35,36^. Here, brain features are associated one by one with the target phenotype. Those with the strongest univariate effect sizes are selected for modelling, while all others are discarded. Although feature selection can reduce training time, improve predictions and simplify interpretability, it may overlook weaker, neurobiologically meaningful signals.

Traditionally, selected features are interpreted as the neurobiology underlying individual variation in the predicted phenotype. For instance, models are often named after the phenotype they predict (for example, ‘working memory network’ or ‘depression network’). By treating selected features as definitive, these practices give the impression that a model uniquely represents that phenotype’s neural correlates. Implicitly, unselected features are overlooked as less important—or, in some cases, inconsequential—to model performance and neurobiology.

As a result, feature selection may risk misrepresenting the neurobiology underlying individual differences in behaviour. Associations between the brain and real-world phenotypes are probably best characterized by widely distributed neural circuits with small effect sizes^22,37,38^. Conventional univariate approaches, including univariate feature selection, may permit only the most robust and straightforward associations—the tip of the iceberg—to survive^39^. Yet, recent work demonstrates that features with weaker univariate effect sizes can often be combined to yield prediction accuracy comparable to that of more strongly associated features^11^. Feature selection may thus oversimplify the complex neurobiology subserving behaviour. If features discarded during selection support meaningful predictions, then restricting interpretation to top-ranked features truncates neurobiological complexity. Despite the widespread use of univariate feature selection, how it balances the trade-off between simplification for optimizing modelling and oversimplification that misrepresents true neurobiology remains understudied.

Here we test whether unselected features are meaningful for prediction and neurobiological interpretation. Our analyses span 12,200 participants across four large-scale neuroimaging datasets, both functional and structural connectivity, and 13 outcomes, including age, sex, cognitive abilities, developmental measures and psychiatric phenotypes. We use an original prediction paradigm wherein connectome features are divided into non-overlapping subsets (that is, an edge can be in only a single subset) on the basis of their association with a target phenotype. We show that multiple subsets overlooked by feature selection can yield significant prediction accuracies but diverging biological interpretations. These results suggest that focusing on the ‘top’ features may paint an incomplete picture of the underlying neurobiology and reinforce that subtle brain-wide signals should not be ignored. They also suggest that multiple neurobiologically distinct models may exist for a given phenotype, which could have important implications for identifying meaningful subtypes within clinical or research populations.

## Results

### Overview

We used four developmental neuroimaging datasets: the Healthy Brain Network (HBN, *n* = 1,110) dataset^40^, the Adolescent Brain Cognitive Development (ABCD, *n* = 9,371) Study^41^, the Human Connectome Project in Development (HCPD, *n* = 428) dataset^42^ and the Philadelphia Neurodevelopmental Cohort (PNC, *n* = 1,291) dataset^43^. Details about the datasets are presented in Supplementary Table 1 and in the Methods; see also Adkinson et al.^24^ for further context. Our first set of analyses focused on connectome-based predictive modelling (CPM), which explicitly relies on univariate feature selection. We later present complementary results using ridge regression (which can handle correlated features without feature selection) with and without feature selection.

**Fig. 1:**
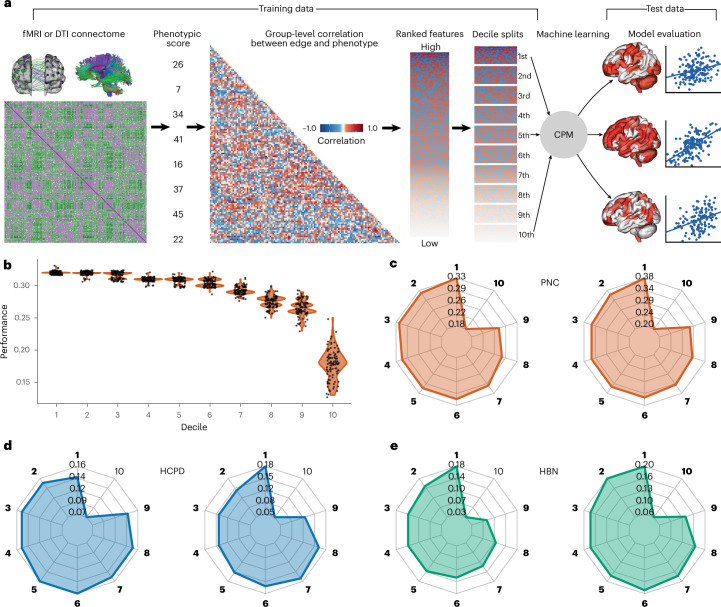
CPM across non-overlapping decile-ranked brain connectivity features. **a**, Workflow illustrating the decile-based CPM pipeline, including the initial correlation of connectivity features with phenotypic outcome, ranking features on the basis of group-level correlations between edges and phenotype, splitting features into deciles, and evaluating each decile-based model. DTI, diffusion tensor imaging. **b**, Violin plot showing the predictive performance of models trained on each decile of features within the PNC dataset for executive function. **c**–**e**, Radar plots depicting predictive performance across deciles for PNC executive function (**c**, left), PNC language abilities (**c**, right), HCPD executive function (**d**, left), HCPD language abilities (**d**, right), HBN executive function (**e**, left) and HBN language abilities (**e**, right). Bold decile numbers indicate significant predictions.

We modified CPM^44^ such that connectome features were divided into ten non-overlapping deciles on the basis of the strength of their association with a target phenotype (Fig. 1a). Specifically, to prevent data leakage, we computed the Pearson correlation coefficient between every connectivity edge and the target phenotype in the training set. Features were then ranked in descending order on the basis of the absolute value of their correlation coefficients. Following ranking, the complete set of connectivity features was partitioned into ten non-overlapping deciles, with the first decile comprising the top 10% of features (that is, those most likely included in established neuroimaging predictive modelling pipelines) and the last decile comprising the bottom 10% of features. Each decile was subsequently used to predict the phenotype of interest in the respective test set. Models were trained using 100 iterations of tenfold cross-validation. Model performance was evaluated with Pearson’s correlation (*r*), representing the correspondence between predicted and actual behavioural scores, along with the cross-validation coefficient of determination (*q*^2^) and mean square error (MSE). Significance was assessed using permutation testing with 1,000 iterations of randomly shuffled behavioural data labels. As in previous work, the Haufe transform was not applied to the CPM models^33,45^.

### Prediction accuracy is not exclusive to top-ranked features

We generated models of language abilities and executive function in the PNC, HBN and HCPD datasets using our modified CPM paradigm. As in previous work^24^, we used principal component analysis (PCA) to combine individual cognitive measures into latent variables of executive function and language abilities. Models constructed from the first through ninth feature deciles successfully predicted executive function and language abilities across the PNC, HBN and HCPD (Fig. 1 and Supplementary Figs. 1–3). Lower-ranked features commonly overlooked during model building continued to demonstrate significant prediction performance. For example, for PNC executive function, the first decile achieved a prediction performance of *r* = 0.33 (*P* = 0.001, *q*^2^ = 0.09, MSE = 1.24), while the second (*r* = 0.32, *P* = 0.001, *q*^2^ = 0.08, MSE = 1.24) through sixth (*r* = 0.31, *P* = 0.001, *q*^2^ = 0.07, MSE = 1.25) deciles achieved significant predictions. Notably, while these models perform similarly, the underlying edge features are non-overlapping, in that a given edge could only be in one decile. Additionally, the first decile did not always exhibit the best prediction performance. For HCPD executive function, the fifth decile (*r* = 0.16, *P* = 0.001, *q*^2^ = −0.07, MSE = 2.08) numerically outperformed deciles with features ranked as more highly informative (for example, first decile, *r* = 0.14, *P* = 0.002, *q*^2^ = −0.10, MSE = 2.14). Overall, the first decile showed no significant differences in prediction performance compared to the second (*t*_5_ = −1.964; *P* = 0.107; Cohen’s *d* = 0.802; 95% confidence interval (CI), (−0.035, 0.005)), fifth (*t*_5_ = −2.331; *P* = 0.067; Cohen’s *d* = 0.953; 95% CI, (−0.042, 0.002)), sixth (*t*_5_ = −1.939; *P* = 0.110; Cohen’s *d* = 0.791; 95% CI, (−0.043, 0.006)) and seventh (*t*_5_ = 0.904; *P* = 0.408; Cohen’s *d* = 0.369; 95% CI, (−0.452, 0.942)) deciles (Supplementary Table 2). Performances for all deciles are presented in Supplementary Table 3.

### Overlooked feature sets also pass external validation

**Fig. 2:**
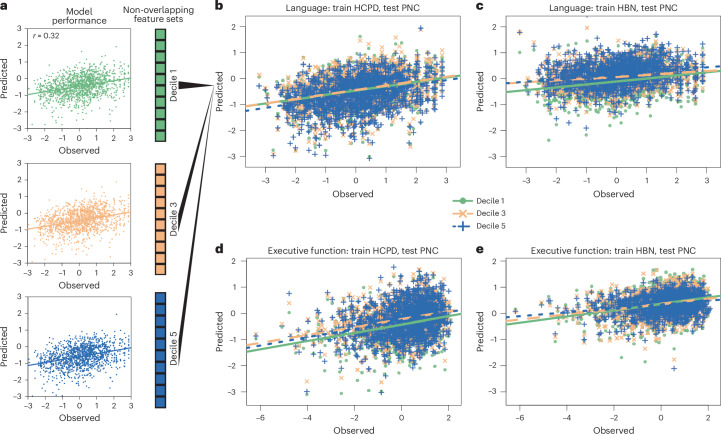
Multiple non-overlapping feature sets are generalizable to external datasets. **a**, Observed versus predicted values for language abilities predictions when training in the HCPD and testing in the PNC using the first (green), third (orange) and fifth (blue) decile feature subsets. **b**–**e**, Scatter plots for the first (green), third (orange) and fifth (blue) decile feature subsets overlaid to enable direct comparison of predictive accuracy across deciles. Results are shown for training a language abilities model in the HCPD and testing in the PNC (**b**), training a language abilities model in the HBN and testing in the PNC (**c**), training an executive function model in the HCPD and testing in the PNC (**d**) and training an executive function model in the HBN and testing in the PNC (**e**).

As external validation is the gold standard for model evaluation^23^, we applied our decile-based models to three independent datasets. Despite the absence of overlapping features between deciles, deciles beyond the first demonstrated successful external validation for executive function and language abilities across all 12 cross-dataset prediction scenarios (Fig. 2 and Supplementary Tables 4–6). For example, PNC executive function models tested in the HCPD had no significant differences in external validation between deciles 1 through 9. In other words, the PNC executive function models from the ninth decile (*r*_426_ = 0.13; *P* = 0.004; Cohen’s *d* = 0.262; 95% CI, (0.035, 0.220)) generalized to the HCPD just as well as those from the first decile (*r*_426_ = 0.14; *P* = 0.002; Cohen’s *d* = 0.283; 95% CI, (0.046, 0.230)). Similar trends were observed for other models. Models tested in the PNC (*r* generally >0.25; Fig. 2) and HCPD (*r* generally >0.10) outperformed those tested in the HBN (*r* generally <0.10).

### Overlooked features yield diverging neurobiological interpretations

**Fig. 3:**
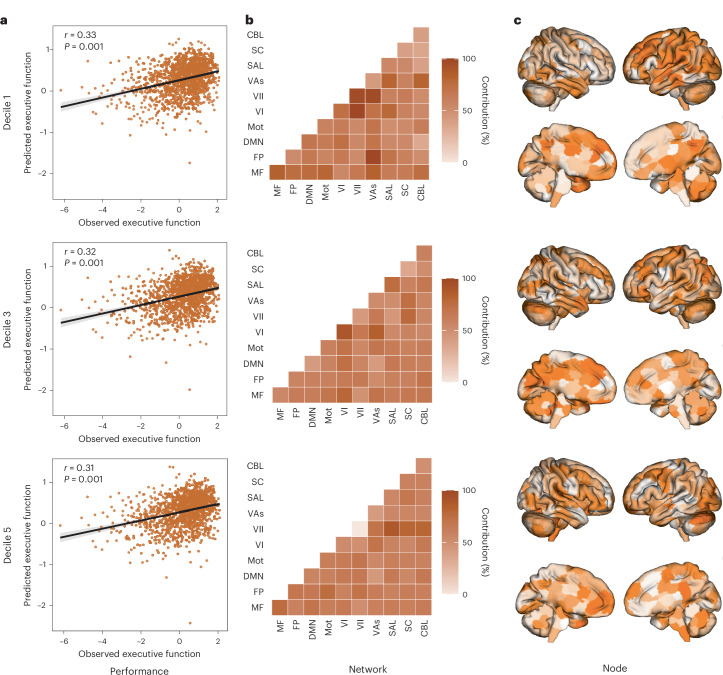
Overlooked feature sets offer similar prediction performance but unique neuroanatomical contributions. **a**, Model performances across deciles as measured by the Pearson correlation between observed and predicted values. The grey bands show the 95% CIs. One-sided *P* values are reported. **b**, Canonical network contributions to PNC executive function predictions. Contributions represent the number of selected features averaged across folds (that is, the proportion of folds in which each edge was selected in the median-performing model) grouped by canonical functional network pairs. Diagonal cells represent contributions of edges within a single network; off-diagonal cells represent contributions of edges between networks. The values were normalized by the number of edges in each network group. Darker colours indicate higher relative contributions. MF, medial frontal; FP, frontoparietal; DMN, default mode; Mot, motor cortex; VI, visual A; VII, visual B; VAs, visual association; SAL, salience; SC, subcortical; CBL, cerebellum. **c**, Node-level contributions to PNC executive function predictions. Node contributions show the sum of all edgewise ridge regression coefficients averaged across folds.

Next, because models performed similarly across non-overlapping feature subsets, we sought to determine whether similar brain features were implicated in each decile-based model by comparing within- and between-network functional connectivity. The canonical networks underlying predictions differed between deciles for both executive function and language abilities (Fig. 3 and Supplementary Fig. 4). For example, for PNC executive function, connectivity between the visual association and frontal-parietal networks was prominent in decile 1 but less informative for deciles 2 through 5 (Fig. 3b). Networks from different deciles showed weak to moderate explained variance (average 13%; Supplementary Fig. 5), with only one network pair explaining greater than 50% variance. Explained variance between networks from neighbouring deciles was greater than explained variance between those from non-neighbouring deciles. For PNC executive function, the explained variances were 0.5% between deciles 1 and 3, 3% between deciles 1 and 4, and 4.5% between deciles 1 and 5 (Supplementary Fig. 5). That networks became increasingly dissimilar with further deciles was consistent across datasets and phenotypes. Trends were also similar when shifting from a network-based comparison to a node-based approach, evaluating features on the basis of the sum of model contributions for each of the 268 nodes (Fig. 3c).

The selected features were more frequently present across folds in the higher deciles than in the lower deciles (Supplementary Table 7). On average across all models, relative to the first decile, 84.64% of second-decile edges, 38.82% of third-decile edges and 22.19% of fifth-decile edges were present (that is, selected) in at least five of the ten cross-validation folds. To account for this variation, the proportion of folds in which each edge was selected for CPM models was used for all interpretability analyses (Methods). Analyses were repeated using edges selected consistently across at least five folds, yielding similar results (Supplementary Fig. 6).

### The utility of overlooked features generalizes to psychiatric, developmental and demographic phenotypic domains

**Fig. 4:**
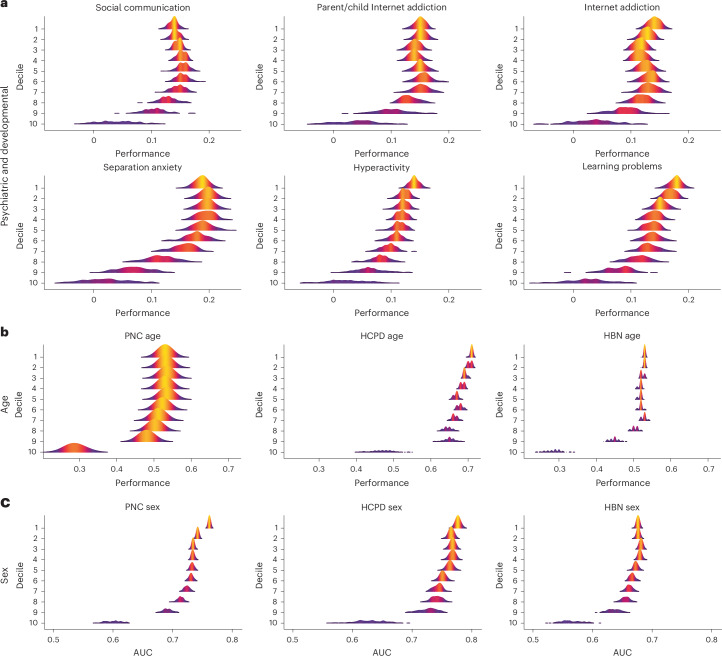
Decile-based predictive modelling performance across psychiatric, developmental and demographic phenotypes in the HBN. **a**, Ridgeline plots showing the distribution of prediction performances (Pearson’s *r*) across 100 iterations for Social Communication Questionnaire, Strengths and Difficulties Questionnaire Hyperactivity, Screen for Anxiety Related Disorders Separation Anxiety, Parent-Child Internet Addiction Test, Internet Addiction Test and Conners 3 Self-Report Learning Problems scores in the HBN. **b**, Predictive performance of age for the PNC, HCPD and HBN datasets. **c**, Predictive performance of sex in the PNC, HCPD and HBN datasets. AUC, area under the curve.

We next examined whether overlooked features have relevance in domains beyond language and executive function. First, predictions were performed within a diverse selection of developmental and psychiatric phenotypes in the HBN dataset (*n* = 747 participants; Methods). For every phenotype, deciles beyond the first retained predictive utility (Fig. 4a). Notably, for the Social Communication Questionnaire^46^, the second through seventh deciles achieved numerically greater prediction performance (*r* = 0.15 to 0.16, MSE = 19.61 to 19.73, *q*^2^ = −0.02) than the first decile (*r* = 0.14, *q*^2^ = −0.03, MSE = 19.80). Predictive models of cognitive, developmental and psychiatric phenotypes generally exhibit low effect sizes. We therefore also tested whether our findings extend to predictions of larger-effect-size phenotypes by predicting age and sex in the PNC, HCPD and HBN. Once again, traditionally overlooked feature sets retained significant predictive capabilities for sex and age in all three datasets (Fig. 4b,c).

### Overlooked features with ridge regression

We then repeated several analyses using ridge regression. Ridge regression can handle high-dimensional data without the need for feature selection^47^, although feature selection is still commonly employed with ridge regression. Ridge regression was performed both without feature selection and with using an analogous decile-based feature selection approach as for the previous results. For biological interpretation, the Haufe transform^48^ was applied to the model weights.

Feature selection increased prediction performance and decreased training time for ridge regression. Ridge regression models without feature selection performed significantly worse than the first decile models (*t*_5_ = 3.230; *P* = 0.023; Cohen’s *d* = 1.319; 95% CI, (0.013, 0.114)) and similar to the second (*t*_5_ = 2.500; *P* = 0.055; Cohen’s *d* = 1.021; 95% CI, (0.001, 0.068)) and third decile models (*t*_5_ = 0.466; *P* = 0.661; Cohen’s *d* = 0.190; 95% CI, (−0.015, 0.022); Supplementary Tables 8 and 9). Models from all other deciles performed worse. These results highlight the benefit of feature selection, even when methods do not explicitly rely on it.

**Fig. 5:**
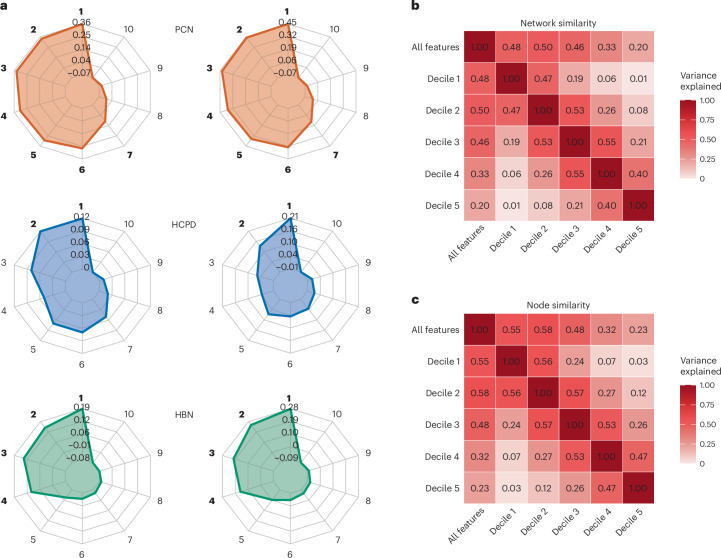
Decile-based model performance using ridge regression. **a**, Radar plots depicting executive function (left) and language abilities (right) model performance across deciles for the PNC (orange), HCPD (blue) and HBN (green). Bold decile numbers indicate prediction performance *r* ≥ 0.10. **b**, Pairwise explained variance between models at the network level averaged across datasets. **c**, Pairwise explained variance between models at the node level averaged across datasets. Higher explained variance reflects greater similarity in features across deciles. Supplementary Fig. 7 presents the results for individual datasets and phenotypes.

Similar to CPM, models beyond the first decile exhibited significant predictions (Fig. 5a). Unlike CPM, however, prediction performance for the subsequent deciles was worse than for the previous deciles. For example, models from the first decile outperformed models from the second decile (*P* = 0.048). Models based on non-overlapping deciles exhibited complementary connectivity patterns from each other and models without feature selection. Decile-based models typically explained less than 40% (at the network level) and 43% (at the node level) of the variance of the models without feature selection (Fig. 5b,c). Only three decile-based models explained greater than 50% of the variance of the models without feature selection. In two thirds of the comparisons, the most similar models to those without feature selection were not from the top-decile model (Supplementary Fig. 7). In other words, models built on all features are (on average) more similar to lower-decile models.

### Replication using partial correlation

Functional MRI (fMRI) features are known to have high degrees of spatial and temporal autocorrelations^49,50^. To isolate direct connections by removing shared variance between nodes, we repeated our analyses using functional connectivity matrices generated with partial correlation instead of Pearson’s correlation in the PNC dataset. The partial correlation connectome results resembled those yielded by diffusion-based connectomes (described below) in that performances decayed more monotonically relative to the standard PNC functional connectomes (Supplementary Tables 10 and 11).

### Overlooked features are also useful in other imaging modalities

We used diffusion-weighted imaging data from the ABCD study to test whether the predictive utility of lower deciles is conserved across imaging modalities. Structural connectomes were generated on the basis of each fibre’s average quantitative anisotropy value connecting two end regions (Methods). We created functional and structural models for the 6,271 ABCD participants with both fMRI and diffusion data to predict NIH Toolbox age-corrected fluid, crystallized and composite intelligence scores.

**Fig. 6:**
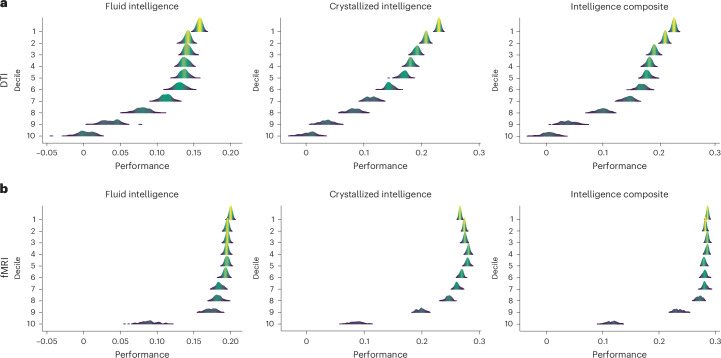
Decile-based predictive modelling performance across diffusion tensor imaging and fMRI data in ABCD. **a**,**b**, Ridgeline plots showing the distribution of prediction performances (Pearson’s *r*) across 100 iterations for NIH Toolbox age-corrected fluid, crystallized and composite intelligence scores using diffusion (**a**) and fMRI (**b**) data.

Like functional models, structural models retained considerable predictive abilities throughout lower-decile feature sets (Fig. 6). Diffusion-based models showed a steady, linear decline in predictive performance across deciles, with each decile performing slightly worse than the previous one. This differs from functional data, which maintained relatively stable performance across most deciles, with a sharp drop-off occurring in the lowest deciles. More broadly, functional connectomes generally achieved higher prediction performances than structural models for each phenotype across deciles. Results based on diffusion tensor imaging were replicable when using the entire ABCD sample (*n* = 9,371; Supplementary Fig. 8) and when predicting a phenotype of higher effect size in the HBN dataset (age, *n* = 767; Supplementary Fig. 9).

### Sensitivity and exploratory analyses

To further evaluate the robustness of our findings, we conducted a series of sensitivity analyses using alternative feature selection and modelling approaches. First, we examined whether the observed predictive performances persisted when using alternative feature groupings. Instead of dividing features into ten deciles, we partitioned them into percentiles (1% subsets), ventiles (5% subsets) and quintiles (20% subsets) on the basis of their correlation with the target phenotype (Supplementary Tables 3, 12 and 13). We also tested whether partitioning features on the basis of alternative statistical metrics influenced the results. Features were grouped into non-overlapping bins on the basis of their significance (*P* values) or effect size (Pearson’s *r* values) with the target phenotype (Supplementary Fig. 10). We then evaluated prediction performance while controlling for the effects of the non-interest variables age, sex, racial/ethnic minority representation, socio-economic status and head motion (Supplementary Table 14). To test the impact of incorporating task fMRI into averaged connectomes on model performances, we repeated the PNC analyses using only resting-state connectomes (Supplementary Table 15). In each scenario, feature subsets beyond those that are most highly ranked demonstrated significant predictions. Preliminary results also suggest that the top-ranked decile was not always the best-fit model for certain subsets of individuals (Supplementary Fig. 11).

## Discussion

Here we evaluated whether unselected features can meaningfully contribute to building and interpreting predictive models in neuroimaging. Using a decile-based feature ranking paradigm, we demonstrated that significant predictive capabilities are not exclusive to top-ranked connectivity features. Across functional and structural connectivity data and a wide range of phenotypes and datasets, lower-ranked, unselected features achieved significant prediction accuracy often comparable to that of traditionally selected features (that is, the most highly ranked features). Lower-ranked features revealed complementary connectivity patterns that often provided unique neuroanatomical contributions. Overall, the phenomenon in which multiple feature sets can achieve significant predictions could lead to divergent neurobiological interpretations of brain–behaviour associations. As such, while feature selection may improve many aspects of predictive modelling, it may present only the tip of the iceberg when certain disregarded features may be just as meaningful.

Our results are aligned with several emerging neuroimaging trends. First, they support the growing appreciation of widely distributed brain networks, as opposed to more localized or simplified networks. Evidence from human^39,51–53^ and animal^54,55^ studies demonstrates that the whole brain responds to stimuli, supporting the view that networks—not single nodes—drive behaviour and cognition. Because brain–behaviour models depend on widely distributed networks, even edges with weak individual correlations to behaviour may play a meaningful role when considered as part of a broader functional network. While sparser models achieved by feature selection are convenient, they may oversimplify the underlying neurobiology of individual variation in behaviour.

Next, while predictive models are more replicable than brain-wide association studies^3,7^, replicability challenges still exist^5,56,57^. It is not uncommon for multiple cases of the same phenotype to implicate different neurobiological processes^18^. Our findings may help explain a portion of this variability. In certain instances of replication failure, the different networks identified across studies may represent select snapshots of a more complex neurobiological landscape rather than true disagreement. This concept aligns with the growing trend towards transparent visualizations, which emphasizes retaining and displaying all data rather than discarding sub-threshold results^32,58^. Thresholded representations emphasize differences while obscuring underlying similarities.

Our results also align with recognized differences between multivariate and univariate analyses in neuroimaging^1,59^. For example, univariate features in the connectome (that is, edges) tend to have poor reliability^60,61^. However, multivariate reliability methods (for example, fingerprinting and discriminability) show comparably high reliability of connectomes^62–67^. This increased reliability reflects the high-dimensional structure of connectomes. Similarly, selecting a single feature at a time without considering the other features ignores this high-dimensional variance structure^68^. Such approaches are emerging but remain uncommon^69,70^. Alternatively, approaches that create and select the predictive features, such as deep learning, may be paths forward^71,72^. Overall, the limitations of univariate feature selection should be kept in mind when considering the strengths of predictive modelling.

On the surface, it may appear contradictory that higher- and lower-ranked features can predict outcomes similarly. This result is probably explained by the high degree of spatial and temporal autocorrelation among brain features^49,50^. When a new feature is added to a model, accuracy will increase only if the new feature has information that is unique from the existing features. Two features that are highly correlated with a phenotype will probably have a high correlation between them. Pooling lower-ranked (but uncorrelated) features may thus perform as well as or better than pooling highly ranked (but highly correlated) features. Pooling features can also enhance robustness by suppressing noise, even if their individual univariate associations are weak. In other words, lower signal-to-noise ratios turn relatively strong aggregate brain–behaviour associations into weak univariate associations. Alternatively, predictive models can inadvertently capture information that is not of interest, such as confounds^17,73^, and they are known to perform more poorly for individuals who defy sample stereotypes^16^. Lower-ranked features may be more susceptible to learning from such information. The predictions from lower-ranked features may therefore reflect these properties rather than the underlying neurobiology.

The results from our structural models largely aligned with those generated from functional connectomes; however, while functional model performances were relatively stable until the lowest deciles, diffusion-based model performances showed a more steady, linear decline across deciles. Given that functional connectomes achieved higher prediction performances, one possibility is that the utility of lower deciles persists longer for higher-effect-size predictions. This pattern could suggest that high-effect-size phenotypes are distributed more broadly across the brain, or alternatively, that machine learning is more effective at capturing phenotypes with a larger number of contributing features. However, our initial investigation using age prediction with structural connectomes suggests that this explanation does not hold. An alternative explanation could relate to the low signal-to-noise ratio and high temporal autocorrelation of fMRI time series data, which might yield signal redundancies across deciles and account for why performance remains similar across several deciles. Indeed, preliminary results using functional connectomes generated using partial correlation exhibited a similar pattern of performance decay to that observed with diffusion connectomes. Nevertheless, considerations discussed for functional models probably hold for structural models.

Identifying and defining biologically meaningful clusters (for example, subtypes) of individuals within a dataset is of great interest within the field^74–77^. Our results suggest that multiple neurobiologically distinct models may capture a given phenotype, each requiring different models^16,78^, which has important implications for these ongoing efforts^79–88^. For example, exploratory analyses indicate that one subtype may be best predicted by decile 1, while another subtype may be best predicted by decile 2. If this is the case, the top-ranked feature set identified by a particular analysis could be influenced by the relative proportions of subtypes in the sample. As the distribution of subtypes shifts, the features deemed most predictive could also change. While our preliminary results point to this possibility, further analysis is needed to validate the extent to which subtype composition affects which features are selected. Future work could leverage more formal clustering approaches to identify latent subgroups and evaluate whether these align with distinct feature subsets^89^.

Similarly, unselected, lower-ranked features may be new targets for emerging interventions and strategies for individualized medicine. Traditionally, the highest-ranked features from a predictive model are selected as potential targets^31^, though these features may not match a specific therapy. For example, it is difficult to stimulate subcortical regions with transcranial magnetic stimulation^90,91^. If a model consists of these regions, it would be a poor fit to inform transcranial magnetic stimulation targets. However, overlooked, lower-ranked features may be more anatomically accessible (for example, cortical versus subcortical) and still predict the phenotype of interest with the same accuracy as the high-ranked features. Looking beyond the traditional way of selecting features may better align predictive models with therapeutic approaches. Still, it remains unclear which features are the best targets for intervention. Brain features that predict inter-individual variation in symptoms or treatment outcomes are not necessarily the same as those causally involved in symptom manifestation^92,93^.

A strength of this study lies in the comprehensive validation of our models across diverse datasets and methodological frameworks. We leveraged four large-scale neuroimaging datasets to enhance statistical power and generalizability. The PNC, HBN, HCPD and ABCD datasets exhibit notable variability in key aspects of study design including recruitment geography, behavioural assessment, imaging acquisition, participant demographics and clinical symptom burden^24^. The utility of commonly disregarded features survived external validation, demonstrating the resilience of our models to differences in participant demographics and study design. Neuroimaging predictive models will achieve real-world utility only if they can overcome these dataset-specific idiosyncrasies (that is, “dataset shifts”)^24,56,94–96^. Finally, we also performed an extensive set of sensitivity analyses. Our results were robust to both functional and structural imaging modalities and across a range of cognitive, psychiatric and developmental phenotypic domains.

We used Pearson’s correlation as our main measure of prediction performance. However, many models exhibited negative *q*^2^, indicating that the sample mean achieves a lower MSE. On the basis of *q*^2^, these models did not successfully predict outcomes. Nevertheless, models with negative *q*^2^ can still have high utility. For example, such models may accurately preserve the relative ranking of individuals, which can be valuable for clinical tasks involving risk stratification and prioritization of support or intervention. While a single measure of prediction performance can never fully characterize a model’s performance, our interpretations are based on a single measure, Pearson’s correlation. We present multiple measures to provide a more comprehensive view of the model. Though many models may no longer exhibit significant prediction with *q*^2^, the main results of the paper still hold.

There were several limitations to our study. First, we pursued a network-centric approach focusing on the connectivity strengths (that is, edges) between 268 brain regions (that is, nodes) as features. Node-based models, which may capture local brain dynamics or region-specific activity patterns that are obscured in connectome-based approaches, could reveal different results. However, connectome-based models are more frequently employed in the field, in part due to superior predictive abilities^97^. Second, it is unclear whether overlooked features maintain their predictive utility across phenotypes that are hypothesized to rely on more circumscribed functional networks, such as reaction time. Finally, this investigation relies on developmental datasets exclusively from the USA. Future work should investigate the utility of commonly overlooked features in adult populations and those from non-Western countries^98,99^.

Collectively, our findings highlight that commonly overlooked features may possess comparable predictive and neurobiological significance. They support the growing appreciation of widely distributed brain networks, as opposed to more localized or simplified networks. Our findings also point to the potential presence of subtypes in the connectivity data, wherein different sets of features represent the best model for different groups of individuals. Overall, better understanding and characterization of overlooked features will help improve the generalizability of predictive models.

## Methods

### Datasets

The PNC participants were 1,291 individuals ages 8–21 recruited from the greater Philadelphia, Pennsylvania, area^43^. All imaging was performed on a single scanner at the Hospital of the University of Pennsylvania. The participants completed rest, emotion task and *N*-back task fMRI runs^100^. The measures of language abilities were the Penn Verbal Reasoning Task from the Penn Computerized Neurocognitive Battery and the total standard score from the Wide Range Assessment Test Reading Subscale^101,102^. The executive function measures were the Letter N-Back, Conditional Exclusion and Continuous Performance tasks from the Computerized Neurocognitive Battery.

The HBN participants were 1,110 individuals ages 6–17 recruited from the New York City, New York, region^40^. Imaging for this multi-site study was performed via the HBN mobile MRI scanner in Staten Island and at the Rutgers University Brain Imaging Center, the CitiGroup Cornell Brain Imaging Center and the CUNY Advanced Science Research Center. The participants completed two rest fMRI runs as well as ‘Despicable Me’ and ‘The Present’ movie-watching scan sessions. The measures of language abilities were the Elision, Blending Words, Nonword Repetition, Rapid Digit Naming and Rapid Letter Naming scaled scores from the Comprehensive Test of Phonological Processing and the Phonemic Decoding Efficiency, Sight Word Efficiency and Total Word Reading Efficiency scaled scores from the Test of Word Reading Efficiency^103,104^. The executive function measures were the Flanker Inhibitory Control and Attention, List Sorting Working Memory, Pattern Comparison Processing Speed and Dimensional Change Card Sort age-corrected standard scores from the NIH Toolbox^105^.

To select developmental and psychiatric phenotypes of meaningful effect size, we first performed predictions using the original CPM framework. We identified scales and subscales with prediction performance *r* > 0.10 by testing predictions on the 575 HBN participants who had complete data for all 71 measures. This resulted in 13 scales and subscales. We repeated the predictions using the 747 participants who had data available for all 13 scales. Of these, 6 scales achieved prediction performances of *r* ≥ 0.15, which were used for our decile-based prediction paradigm: the Social Communication Questionnaire^46^ score, the Hyperactivity Scale score from the Strengths and Difficulties Questionnaire^106,107^, the Separation Anxiety score from the Screen for Anxiety Related Disorders^108^, the Learning Problems score from the Conners 3 Self-Report^109^, and the Internet Addiction Test and Parent-Child Internet Addiction Test^110^ scores.

The HCPD participants were 428 individuals ages 8–22 recruited from St. Louis, Missouri; Twin Cities, Minnesota; Boston, Massachusetts; and Los Angeles, California^42^. Imaging for this multi-site study was performed at the University of Minnesota, Washington University in St. Louis, Harvard University and the University of California, Los Angeles. The participants completed rest fMRI runs^111^. The measures of language abilities were the Picture Vocabulary and Oral Reading Recognition age-corrected standard scores from the NIH Toolbox. The executive function measures were the Flanker Inhibitory Control and Attention, List Sorting Working Memory, Pattern Comparison Processing Speed, Dimensional Change Card Sort and Picture Sequence Memory age-corrected standard scores from the NIH Toolbox.

The ABCD participants were 9,371 individuals ages 9–10 recruited from 21 sites across the USA: Children’s Hospital Los Angeles, Florida International University, Laureate Institute for Brain Research, Medical University of South Carolina, Oregon Health & Science University, SRI International, UC San Diego, UCLA, University of Colorado Boulder, University of Florida, University of Maryland at Baltimore, University of Michigan, University of Minnesota, University of Pittsburgh, University of Rochester, University of Utah, University of Vermont, University of Wisconsin–Milwaukee, Virginia Commonwealth University, Washington University in St. Louis and Yale University^41,112^. The cognitive measures were the Fluid Intelligence, Crystalized Intelligence and Intelligence Composite age-corrected scores from the NIH Toolbox.

### fMRI data preprocessing

In all datasets, the data were motion-corrected. Additional preprocessing steps were performed using BioImage Suite^113^. This included regression of covariates of no interest from the functional data, including linear and quadratic drifts, mean cerebrospinal fluid signal, mean white matter signal and mean global signal. Additional motion control was applied by regressing a 24-parameter motion model, which included six rigid body motion parameters, six temporal derivatives and the squares of these terms, from the data. Subsequently, we applied temporal smoothing with a Gaussian filter (approximate cut-off frequency, 0.12 Hz) and grey matter masking, as defined in common space. The Shen 268-node atlas was then applied to parcellate the denoised data into 268 nodes^114^. Finally, we generated functional connectivity matrices by correlating each node time series data pair and applying the Fisher transform.

Data were excluded for poor data quality, missing nodes due to the lack of full brain coverage, high motion (>0.2 mm mean frame-wise motion) or missing behavioural/phenotypic data. Each participant’s connectome included all available resting-state and task fMRI data with low motion (<0.2 mm). Connectomes for individual conditions (that is, resting-state and task) were created independently and then averaged. Combining connectomes across fMRI data improves reliability and predictive power^14,115^. Participants without one low-motion fMRI run were excluded. For the PNC, 246 participants were excluded due to image quality or motion, and 61 participants were excluded due to incomplete phenotypic data. For the HBN, 1,387 participants were excluded due to image quality or motion, and 829 participants were excluded due to incomplete phenotypic data. For the HCPD, 57 participants were excluded due to image quality or motion, and 167 participants were excluded due to incomplete phenotypic data. For ABCD, 342 participants were excluded due to incomplete phenotypic data.

### ABCD diffusion data preprocessing

FIB files were downloaded from https://brain.labsolver.org. As described at https://brain.labsolver.org/, a multishell diffusion scheme was used, and the *b*-values were 500, 1,000, 2,000 and 3,000 s mm^−2^. The numbers of diffusion sampling directions were 6, 15, 15 and 60, respectively. The in-plane resolution was 1.7 mm. The slice thickness was 1.7 mm. The diffusion MRI data were rotated to align with the AC-PC line at an isotropic resolution of 1.7 (mm). The restricted diffusion was quantified using restricted diffusion imaging^116^. The diffusion data were reconstructed using generalized *q*-sampling imaging^117^ with a diffusion sampling length ratio of 1.25. The tensor metrics were calculated using diffusion-weighted images with a *b*-value lower than 1,750 s mm^−2^.

As stated in previous work and duplicated here for consistency^118,119^, whole-brain fibre tracking was conducted with DSI-Studio with quantitative anisotropy (QA) as the termination threshold. QA values were computed in each voxel in their native space for each subject and were then used to warp the brain to the template in Montreal Neurological Institute (MNI) space using the statistical parametric mapping nonlinear registration algorithm. Once in MNI space, spin density functions were again reconstructed with a mean diffusion distance of 1.25 mm using three fibre orientations per voxel. Fibre tracking was performed in DSI-Studio with an angular cut-off of 60 degrees, a step size of 1.0 mm, a minimum length of 30 mm, spin density function smoothing of 0.0, a maximum length of 300 mm and a QA threshold determined by the diffusion-weighted imaging signal. Deterministic fibre tracking using a modified FACT algorithm^120^ was performed until 10,000,000 streamlines were reconstructed for each individual. We used the Shen atlas^114^ in MNI space with 268 nodes to construct individual structural connectomes: the pairwise connectivity strength was calculated as the average QA value of each fibre connecting the two end regions and thresholded at 0.001, which resulted in a 268 × 268 adjacency matrix for each participant.

### Creating latent factors of language abilities and executive function

Whether machine learning models are used for real-world prediction or advancing our understanding of neurobiology^121,122^, it is important to overcome dataset-specific idiosyncrasies^1^. The PNC, HCPD and HBN datasets employed disparate measures to assess executive function and language abilities. To harmonize these measures and facilitate direct comparisons across datasets, we used PCA to derive ‘latent’ factors of executive function and language abilities within each dataset. Briefly, as in previous work^24^, PCA was applied to behavioural measures to reduce measurement noise and derive a single composite factor per domain. For the PNC and HBN, PCA was conducted using participants without usable neuroimaging data to maintain independent train and test groups, while for the HCPD, PCA was performed within each fold of cross-validation, using only training data. Behavioural data from participants with imaging data were projected onto the first principal component, which explained 70%, 55% and 77% of the variance in language measures and 53%, 48% and 40% in executive function measures for the PNC, HBN, and HCPD respectively.

### Decile-based CPM

We implemented an original adaptation of the original CPM framework^44^ to evaluate the predictive utility of connectivity features across varying levels of association with the target phenotype. For within-dataset predictions, we performed 100 iterations of a tenfold cross-validation scheme. Within each fold, the model was trained on approximately 90% of the data and tested on the remaining 10% of the data. For each target phenotype, within each fold, we computed the Pearson correlation coefficient between every connectivity edge and the target phenotype across all participants in the training subset. This univariate analysis provided a measure of association strength for each feature. The features were then ranked in descending order on the basis of the absolute value of their correlation coefficients, ensuring that both positive and negative associations were considered equally in the ranking process. Following ranking, the complete set of connectivity features was partitioned into ten non-overlapping deciles. Each decile represented 10% of the total features, with decile 1 comprising the top 10% of features exhibiting the strongest associations with the target phenotype, and decile 10 containing the bottom 10%. Predictions were performed using each decile and tested on the fold’s test set. For sensitivity analyses, independent predictive models were created to account for non-interest variables (age, sex, racial/ethnic minority representation, socio-economic status, head motion and clinical symptom burden) via partial correlation at the step in which brain edges were related to each phenotype^123^.

### Ridge regression CPM

We repeated the analyses using ridge regression CPM to better suit the high-dimensional nature of connectivity data^14^. Specifically, due to the positive semi-definite nature of a functional connectivity matrix, the edges are not independent. Ridge regression is more robust than ordinary least squares in this case. Instead of summing selected edges and fitting a one-dimensional ordinary least-squares model, we directly fit a ridge regression model with training individuals using the selected edges from all the tasks and applied the model to testing individuals in the cross-validation framework. The L2 regularization *λ* parameter was chosen using Bayesian optimization over the training data within each fold. Specifically, MATLAB’s fitrlinear function was used with automatic hyperparameter optimization to select the *λ* value that minimized the cross-validated mean squared error on the training set. All hyperparameter tuning was performed strictly within the training data to avoid information leakage.

### Model performance

Predictive models were trained and tested within each dataset using 100 iterations of tenfold cross-validation. Model performance was evaluated with Pearson’s correlation (*r*), representing the correspondence between predicted and actual behavioural scores. We report mean Pearson’s *r* across the 100 iterations, along with *q*^2^ and MSE (ref. ^5^). Within-dataset plots show data for the median-performing models. To generate null distributions for significance testing, we randomly shuffled the correspondence between behavioural variables and connectivity matrices 1,000 times and reran the CPM analysis with the shuffled data. On the basis of these null distributions, the *P* values for predictions were calculated as in prior work^124^. Only a positive association between predicted and actual values indicates prediction above chance (with negative associations indicating a failure to predict), so one-tailed *P* values are reported. Pearson’s correlation was tested between actual and predicted values to evaluate cross-dataset predictions.

### Model contribution

Predictive networks identified using CPM or ridge regression are composed of multiple brain regions and networks. Each node is defined as a single region in the 268-node Shen atlas, and each node is associated with multiple features (specifically, the edges connecting it to the other 267 nodes). To quantify the contribution of each node and network, we first identified the median-performing model across the 100 iterations for each phenotype. All interpretability analyses were based on this median model.

For ridge regression models, we used the mean of each edge’s coefficient across the ten folds of the median-performing model. The Haufe transform^48^ was applied to model weights. Node contributions were calculated by summing the absolute values of Haufe-transformed weights across all edges connected to a given node. Network-level contributions were computed by averaging the absolute values of Haufe-transformed weights within or between canonical functional networks, normalized by network size. Specifically, to quantify the contribution of each node to a given predictive model, we calculated the *n*th node’s weight summed across all edges (labelled *W*_*n*_) to the model as ${W}_{n}={\sum }_{k=1}^{35,778}{\rm{abs}}({W}_{k})$, for all *k* edges connected to the *n*th node. Next, for the network level, *W*_*k*_ was averaged over each edge within or between canonical functional networks.

For CPM models, edge selection is inherently binary. Each edge is either selected (1) or not (0) in a given fold. We averaged these binary masks across the ten folds of the median-performing model. Node and network contributions were calculated as with ridge, using these fold-averaged values.

### Decile feature comparisons

To quantify the shared variance of model weights across feature deciles, we computed pairwise squared Pearson correlations (*r*^2^) between decile models. For node-level comparisons, ridge regression edge weights were summed for each of the 268 regions defined by the Shen atlas, resulting in a 268-dimensional vector of node-wise importance values per model. The *r*^2^ values between these vectors indicate the proportion of variance in node-level weight distributions shared between models across deciles. For network-level comparisons, edge-wise weights were averaged within each of 55 canonical functional networks, producing a network-level weight vector for each model. Pairwise *r*^2^ values were then calculated between these vectors across deciles, reflecting the degree of similarity in the network-level distribution of feature weights.

For CPM, a similar approach was used, except that binary selection masks (1 for selected and 0 for not selected) were used in place of continuous weights. Node-wise importance vectors were created by summing binary edge selections per node, and network-level vectors were generated by averaging binary selections within each network. Squared correlations (*r*^2^) between these binarized vectors were then calculated across deciles to estimate the similarity of features.

### Reporting summary

Further information on research design is available in the Nature Portfolio Reporting Summary linked to this article.

## Supplementary information

Supplementary Information Supplementary Figs. 1–11 and Tables 1–15.

Reporting Summary

## Data Availability

The data are available through the PNC, HCPD, HBN and ABCD datasets.

## Code availability

The analyses were conducted using MATLAB v.R2024a. The code is available via GitHub at https://github.com/brendan-adkinson/overlooked-features. Preprocessing was carried out using Bioimage Suite v.3.01, which is freely available (https://medicine.yale.edu/bioimaging/suite/). Additional preprocessing was performed with the Human Connectome Project minimal preprocessing pipeline v.3.4.0 (https://github.com/Washington-University/HCPpipelines/releases).
