# Supplementary material for: Feature selection leads to divergent neurobiological interpretations of brain-based machine learning biomarkers
Source: Nat Hum Behav. 2026 Apr 15;10(7):1356–70. doi: 10.1038/s41562-026-02447-y (PMC13388108; doi:10.1038/s41562-026-02447-y)
Supplement: Supplementary file 2 — Reporting Summary [file 41562_2026_2447_MOESM2_ESM.pdf]

nature portfolio

Corresponding author(s): Brendan D. Adkinson

Last updated by author(s): 10/07/2025

Reporting Summary

Nature Portfolio wishes to improve the reproducibility of the work that we publish. This form provides structure for consistency and transparency in reporting. For further information on Nature Portfolio policies, see our Editorial Policies and the Editorial Policy Checklist.

Statistics

For all statistical analyses, confirm that the following items are present in the figure legend, table legend, main text, or Methods section.

n/a	Confirmed	
☐	☒	The exact sample size (*n*) for each experimental group/condition, given as a discrete number and unit of measurement
☐	☒	A statement on whether measurements were taken from distinct samples or whether the same sample was measured repeatedly
☐	☒	The statistical test(s) used AND whether they are one- or two-sided<br>*Only common tests should be described solely by name; describe more complex techniques in the Methods section.*
☐	☒	A description of all covariates tested
☐	☒	A description of any assumptions or corrections, such as tests of normality and adjustment for multiple comparisons
☐	☒	A full description of the statistical parameters including central tendency (e.g. means) or other basic estimates (e.g. regression coefficient) AND variation (e.g. standard deviation) or associated estimates of uncertainty (e.g. confidence intervals)
☐	☒	For null hypothesis testing, the test statistic (e.g. *F*, *t*, *r*) with confidence intervals, effect sizes, degrees of freedom and *P* value noted<br>*Give P values as exact values whenever suitable.*
☒	☐	For Bayesian analysis, information on the choice of priors and Markov chain Monte Carlo settings
☒	☐	For hierarchical and complex designs, identification of the appropriate level for tests and full reporting of outcomes
☐	☒	Estimates of effect sizes (e.g. Cohen's *d*, Pearson's *r*), indicating how they were calculated

Our web collection on statistics for biologists contains articles on many of the points above.

Software and code

Policy information about availability of computer code

Data collection	No data collection was performed in this study. Preprocessing was carried out using Bioimage Suite v3.01, which is freely available (https://medicine.yale.edu/bioimaging/suite/). Additional preprocessing was performed with the Human Connectome Project minimal preprocessing pipeline v3.4.0 (https://github.com/Washington-University/HCPpipelines/releases).
Data analysis	Code for analyses is available on GitHub at https://github.com/brendan-adkinson/overlooked-features.

For manuscripts utilizing custom algorithms or software that are central to the research but not yet described in published literature, software must be made available to editors and reviewers. We strongly encourage code deposition in a community repository (e.g. GitHub). See the Nature Portfolio guidelines for submitting code & software for further information.

Data

Policy information about availability of data

All manuscripts must include a data availability statement. This statement should provide the following information, where applicable:

- Accession codes, unique identifiers, or web links for publicly available datasets
- A description of any restrictions on data availability
- For clinical datasets or third party data, please ensure that the statement adheres to our policy

The following datasets are publicly available but require permission to access. Relevant instructions for data access are available at each individual link. Datasets are available through the Healthy Brain Network Dataset (International Neuroimaging Data-sharing Initiative, https://fcon_1000.projects.nitrc.org/indi/

cmi_healthy_brain_network/), the Adolescent Brain Cognitive Development Study (NIMH Data Archive, https://nda.nih.gov/abcd), the Human Connectome Project Development Dataset (NIMH Data Archive, https://www.humanconnectome.org/study/hcp-lifespan-development/data-releases) and the Philadelphia Neurodevelopmental Cohort Dataset (dbGaP Study Accession: phs000607.v3.p2, https://www.ncbi.nlm.nih.gov/projects/gap/cgi-bin/study.cgi?study_id=phs000607.v3.p2).

Research involving human participants, their data, or biological material

Policy information about studies with human participants or human data. See also policy information about sex, gender (identity/presentation), and sexual orientation and race, ethnicity and racism.

Reporting on sex and gender	Sex representation of each sample is reported among other sample characteristics in Supplementary Table S1.
Reporting on race, ethnicity, or other socially relevant groupings	Racial/ethnic minority representation of each sample is reported among other characteristics in Supplementary Table S1.es.
Population characteristics	Sample population characteristics are presented in Supplementary Table S1.
Recruitment	No participants were collected for this study. Full recruitment procedures for included datasets are available from the Adolescent Brain Cognitive Development Study (Casey et al., 2018), the Healthy Brain Network Dataset (Alexander et al., 2017), the Human Connectome Project Development Dataset (Harms et al., 2018; Somerville et al., 2018), and the Philadelphia Neurodevelopmental Cohort Dataset (Satterthwaite et al., 2014, 2016),
Ethics oversight	The four datasets used in this study were each supervised by their relevant ethical review boards. We have a Yale IRB exemption (HIC: 2000023326) to use public neuroimaging dat

Note that full information on the approval of the study protocol must also be provided in the manuscript.

Field-specific reporting

Please select the one below that is the best fit for your research. If you are not sure, read the appropriate sections before making your selection.

☒ Life sciences ☐ Behavioural & social sciences ☐ Ecological, evolutionary & environmental sciences

For a reference copy of the document with all sections, see nature.com/documents/nr-reporting-summary-flat.pdf

Life sciences study design

All studies must disclose on these points even when the disclosure is negative.

Sample size	We did not pre-determine sample size as we did not collect data. Instead, large, open-source neuroimaging datasets were selected for this study, and all available participants meeting the inclusion criteria outlined in the methods were included.
Data exclusions	For PNC, 246 participants were excluded due to image quality or motion, and 61 participants were excluded due to incomplete phenotypic data. For HBN, 1387 participants were excluded due to image quality or motion, and 829 participants were excluded due to incomplete phenotypic data. For HCPD, 57 participants were excluded due to image quality or motion, and 167 participants were excluded due to incomplete phenotypic data. For ABCD, 342 participants were excluded due to incomplete phenotypic data.
Replication	We demonstrated our findings across several datasets. The methods include internal validation (within-dataset) and external validation (cross-dataset).
Randomization	For within-dataset predictions, the held-out subset was randomly selected across multiple iterations.
Blinding	N/A

Reporting for specific materials, systems and methods

We require information from authors about some types of materials, experimental systems and methods used in many studies. Here, indicate whether each material, system or method listed is relevant to your study. If you are not sure if a list item applies to your research, read the appropriate section before selecting a response.

Materials & experimental systems

n/a	Involved in the study
☒	☐ Antibodies
☒	☐ Eukaryotic cell lines
☒	☐ Palaeontology and archaeology
☒	☐ Animals and other organisms
☒	☐ Clinical data
☒	☐ Dual use research of concern
☒	☐ Plants

Methods

n/a	Involved in the study
☒	☐ ChIP-seq
☒	☐ Flow cytometry
☐	☒ MRI-based neuroimaging

Plants

Seed stocks

N/A

Novel plant genotypes

N/A

Authentication

N/A

Magnetic resonance imaging

Experimental design

Design type

function magnetic resonance imaging; diffusion tensor imaging

Design specifications

Details are published in the relevant papers for the Adolescent Brain Cognitive Development Study (Casey et al., 2018), the Healthy Brain Network Dataset (Alexander et al., 2017), the Human Connectome Project Development Dataset (Harms et al., 2018; Somerville et al., 2018), and the Philadelphia Neurodevelopmental Cohort Dataset (Satterthwaite et al., 2014, 2016).

Behavioral performance measures

Behavioral performance measures are described under the Methods "Datasets" section.

Acquisition

Imaging type(s)

functional (primary analysis); diffusion (secondary analysis of structural connectivity

Field strength

All datasets were collected at 3T, except a portion of the Healthy Brain Network dataset was collected at 1.5T

Sequence & imaging parameters

Details of the sequence and imaging parameters can be found in the relevant papers for the Adolescent Brain Cognitive Development Study (Casey et al., 2018), the Healthy Brain Network Dataset (Alexander et al., 2017), the Human Connectome Project Development Dataset (Harms et al., 2018; Somerville et al., 2018), and the Philadelphia Neurodevelopmental Cohort Dataset (Satterthwaite et al., 2014, 2016).

Area of acquisition

Whole-brain

Diffusion MRI ☒ Used ☐ Not used

Parameters

As described on https://brain.labsolver.org/, a multishell diffusion scheme was used, and the b-values were 500, 1000, 2000, and 3000 s/mm². The number of diffusion sampling directions were 6, 15, 15, and 60, respectively. The in-plane resolution was 1.7 mm. The slice thickness was 1.7 mm. The diffusion MRI data were rotated to align with the AC-PC line at an isotropic resolution of 1.7 (mm). The restricted diffusion was quantified using restricted diffusion imaging 105. The diffusion data were reconstructed using generalized q-sampling imaging 106 with a diffusion sampling length ratio of 1.25. The tensor metrics were calculated using DWI with b-value lower than 1750 s/mm².

Preprocessing

Preprocessing software

Analyses were conducted using Matlab R2024a. Code is available via GitHub at https://github.com/brendan-adkinson/overlooked-features. Preprocessing was carried out using Bioimage Suite v.3.01, which is freely available (https://medicine.yale.edu/bioimaging/suite/). Additional preprocessing was performed with the Human Connectome Project minimal preprocessing pipeline v.3.4.0 (https://github.com/Washington-University/HCPpipelines/releases).

Normalization	Nonlinear normalization into MNI space
Normalization template	MNI304
Noise and artifact removal	Several covariates of no interest were regressed from participants' functional data including linear and quadratic drifts, mean cerebrospinal fluid signal, mean white matter signal, and mean global signal. For additional control of possible motion-related confounds, a 24-parameter motion model (including six rigid body motion parameters, six temporal derivatives, and these terms squared) was regressed from the data. The data were temporally smoothed with a Gaussian filter (approximate cutoff frequency=0.12 Hz).
Volume censoring	Subjects with >0.2 mm mean framewise displacement were excluded.

Statistical modeling & inference

Model type and settings	We employed predictive modeling using a modified connectome-based predictive modeling (CPM) framework. At the first level, we computed univariate Pearson correlations between each brain connectivity edge and the target phenotype within training sets to rank features. Connectivity features were grouped into non-overlapping deciles based on correlation magnitude. At the second level, predictive models were trained separately on each decile using either univariate regression (in standard CPM) or ridge regression (for regularization and to address multicollinearity). Models were evaluated using 10-fold cross-validation repeated 100 times.
Effect(s) tested	We tested the prediction of executive function, language abilities, age, sex, and developmental measures from neuroimaging connectivity data

Specify type of analysis: ☒ Whole brain ☐ ROI-based ☐ Both

Statistic type for inference (See Eklund et al. 2016)	We report the correlation between the observed and predicted phenotype: mean Pearson's r across the 100 iteration, along with the cross-validation coefficient of determination (q2) and mean square error (MSE)
Correction	N/A

Models & analysis

n/a	Involved in the study
☐	☒ Functional and/or effective connectivity
☒	☐ Graph analysis
☐	☒ Multivariate modeling or predictive analysis

Functional and/or effective connectivity	Functional connectivity data was obtained by calculating the Pearson correlation between each pair of parcels and then taking the Fisher transform. The Shen 268 atlas was used (Shen et al., 2013).
Multivariate modeling and predictive analysis	Independent variables consisted of edgewise functional or structural brain connectivity values derived from the Shen 268-node atlas. For each phenotype, we calculated the Pearson correlation between each edge and the target variable in the training set and ranked edges by the absolute value of these correlations. Features were grouped into non-overlapping deciles.<br><br>For modeling, we used connectome-based predictive modeling (CPM), implemented using either univariate or ridge regression to account for multicollinearity among features. Phenotypic outcomes (e.g., language, executive function, developmental measures, age, and sex) were the dependent variables. Executive function and language ability scores were derived using principal component analysis (PCA) to extract latent factors from multiple task-based behavioral measures within each dataset. Model training and evaluation were performed using 10-fold cross-validation, repeated 100 times for robustness. Performance metrics included Pearson's correlation between predicted and actual scores, coefficient of determination (q^2), mean squared error (MSE), and permutation testing for statistical significance.
